# Enhanced recovery after surgery pathway: association with lower incidence of wound complications and severe hypoalbuminemia in patients undergoing posterior lumbar fusion surgery

**DOI:** 10.1186/s13018-022-03070-z

**Published:** 2022-03-24

**Authors:** Shuaikang Wang, Peng Wang, Xiangyu Li, Wenzhi Sun, Chao Kong, Shibao Lu

**Affiliations:** 1 Department of Orthopedics, Xuanwu Hospital, Capital Medical University, No. 45 Changchun Street, Xicheng District, Beijing, China; 2 National Clinical Research Center for Geriatric Diseases, No. 45 Changchun Street, Xicheng District, Beijing, China; 3 Beijing Clinical Research Center for Geriatric Diseases, No. 45 Changchun Street, Xicheng District, Beijing, China

**Keywords:** Enhance recovery, ERAS, Wound complications, Nutrition

## Abstract

**Background:**

Wound complications are associated with worse satisfaction and additional costs in patients undergoing posterior lumbar fusion (PLF) surgery, and the relationship between enhanced recovery after surgery (ERAS) pathway and wound complications remains poorly characterized.

**Methods:**

In this retrospective single-center study, we compared 530 patients receiving ERAS pathway care with previous 530 patients in non-ERAS group. The primary aim of our study was to identify the relationship between the ERAS program and the incidence of postoperative wound-related complications and other complications following PLF surgery; other outcomes included the length of stay (LOS), 90-day hospital and rehabilitation center readmission.

**Results:**

The average patient age was 65 yr. More patients with old cerebral infarction were in ERAS group (*p* < 0.01), and other demographics and comorbidities were similar between groups. Patients in the ERAS group had a lower incidence of postoperative wound-related complications than the non-ERAS group (12.4 vs. 17.8%, *p* = 0.02). The non-ERAS group had a significantly higher rate of wound dehiscence or poor wound healing (6% vs. 3%, *p* = 0.02). ERAS group had a lower incidence of severe postoperative hypoalbuminemia (serum albumin less than 30 g/L) (15.8% vs. 9.0% *p* < 0.01). Additionally, ERAS patients had shorter postoperative LOS (8.0 ± 1.5 vs. 9.5 ± 1.7, *p* < 0.01), lower rate of readmission within 90 days (1.9% vs. 6.4%, p < 0.01) and discharge to rehabilitation center (4.2% vs. 1.0%, *p* < 0.01).

**Conclusion:**

ERAS pathway might help decrease the rates of postoperative wound complications and severe hypoalbuminemia following PLF surgery; additionally, it demonstrated that ERAS pathway was also associated with shorter LOS and lower rate of readmissions within 90 days.

## Background

The incidence of lumbar diseases increases with the rapid population aging in many countries. Posterior lumbar fusion (PLF) surgery is commonly used to treat degenerative lumbar spinal disease [1]. Postoperative wound complications are associated with delayed recovery and reoperation after surgery [2, 3].Wound complications include superficial or deep surgical site infection (SSI), wound dehiscence, poor wound healing and persistent wound drainage [4–6]. Previous literature reported that the rates of SSI following spinal surgery were 0.7–12.0%, and several factors had been reported as risk factors for postoperative wound infection [7–9]. However, few studies investigated the influential factor of other wound healing complications after PLF surgery. Given the higher rate of wound complications, efforts are needed to reduce the incidence of wound complications and enhance recovery. Although there are several known risk factors (including obesity [2], diabetes, operative time [10], larger incision [11]) that are highly associated with wound complications, the impact of perioperative management on wounds complications is less well studied. While the improved surgical techniques are essential for preventing development of complications, the role of perioperative management should not be overlooked.

Enhanced recovery after surgery (ERAS) programs are multidisciplinary perioperative management approaches designed to reduce the surgical stress responses and accelerate recovery after surgery [12]. ERAS protocols have been implemented in other surgical settings for many years. Some studies found that ERAS protocols were associated with reduced length of stay (LOS) and postoperative complications in spinal fusion surgery patients [13, 14]. Previous studies reported that some preoperative and postoperative interventions (e.g., preoperative nutritional support, weight loss and treatment of medical complications) could accelerate recovery and decrease complications, including wound complications [15–17]. Nevertheless, the relationship between ERAS and wound healing remains poorly characterized. Some studies reported that ERAS did not reduce SSI, a finding that is not consistent with others; furthermore, underlying mechanisms for this relationship remain unknown [18, 19].

Therefore, the primary aim of our study was to identify the relationship between an ERAS program and the incidence of postoperative wound-related complications and other complications following PLF surgery. The secondary aim was to evaluate the impact of ERAS pathway on other outcomes, including LOS and 90-day hospital or rehabilitation center readmission.

## Materials and methods

### Study design

This was a single-center retrospective analysis study, and all data were obtained from the electronic medical record system. We included consecutive patients who underwent PLF surgery with or without spinal decompression between January 2017 and July 2021. Patient data included preoperative, intraoperative and postoperative variables. Approval was obtained from the ethics committee of our hospital (permit data 2018.4.3; no. 2018086).

### ERAS protocol

We designed an evidence-based ERAS protocol and implemented the protocol for our perioperative management at our institution from January 2019. The ERAS team consisted of anesthesiologists, spine surgeons, nutritionists and nurses, with two geriatricians and physicians providing valuable suggestions. Our protocol consisted of preoperative, intraoperative, and postoperative interventions based on current, reliable evidence (Table 1). Compliance was evaluated according to the number of achieved individual elements items of the ERAS program by two dedicated staff members who were blinded to patient identify and did not participate in data analysis. The discharge criteria for both groups were as follows: (1) preoperative symptoms were entirely or mostly relieved, or treatment met the patient's expectations; (2) patients were able to walk without any support; (3) patients had no surgery-related complications or the postoperative complications had been controlled; and (4) no further treatment was required.

**Table 1:** Perioperative management pathway of non-ERAS group and ERAS group

	Non-ERAS	ERAS	Compliance
Preadmission	No intervention	1. Education on smoking and excessive drinking cessation; available counseling services at any time; appropriate optimization of chronic disease in outpatient and inpatient settings; nutritional assessment and support	98.3%
Preoperative	Not standardized	2. Informing patients and relatives about risk and discomfort related to procedure in greater detail; ensuring that patients learn and understand ERAS pathway	99.0%
	Route preparation	3. Avoiding mechanical bowel preparation and use of gastric tube	99.4%
	No intervention	4. Drinking oral carbohydrate beverage 2 h before surgery; no prolonged fasting	97.7%
	No intervention	5. Oral administration of 150 mg of Pregabalin	97.1%
Intraoperative	No intervention	6. Infiltration of local anesthesia with a mixture of 10 ml 2% lidocaine and 10 ml 1% ropivacaine into the musculature prior to incision and after skin closure	99.4%
	Antibiotic prophylaxis within 1 h of incision	7. Antibiotic prophylaxis within 1 h of incision	100%
	Not standardized	8. Intravenous infusion of tranexamic acid	98.1%
	Not standardized	9. Maintenance of normothermia; maintaining fluid balance	100%
Postoperative	Intake of fluid on POD1	10. Early intake of fluid on the day of surgery(after recovery from anesthesia)	81.1%
	Not standardized	11. Ealy function rehabilitation on the day of surgery and ambulate on POD1	70.7%
	Not standardized	12. Postoperative prophylaxis against thrombosis and postoperative nausea and vomiting	94.5%
	Not standardized	13. Removal of urinary catheters on the day of surgery and removal of drain tube on POD 2	72.6%
	No intervention	14. Multimodal analgesia and opioid-sparing analgesia	100%
	No intervention	15. Intake of oral nutrition powder for every meal	98.1%
	Route postoperative antimicrobial prophylaxis	16. Route postoperative antimicrobial prophylaxis	100%

POD1: postoperative day 1; POD2: postoperative day 2

### Data collection

**Fig. 1:**
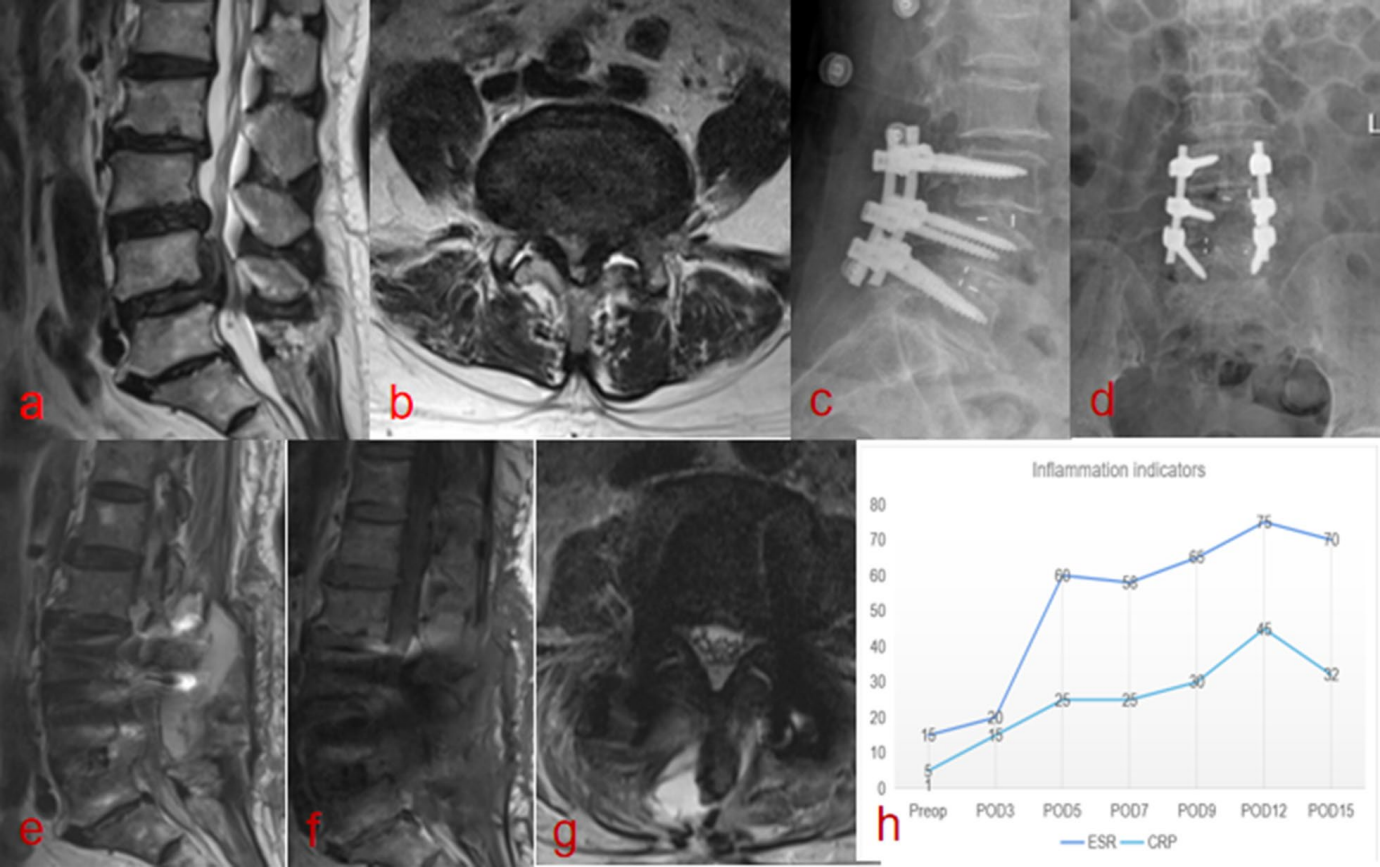
A 57-year-old male patient with postoperative surgical site infection. **a, b** Lumbar MRI showed preoperative disk herniation and spinal canal stenosis. **c, d** Lumbar postoperative X ray showed satisfactory position of screw and cage. **e, f** On the 4th day after surgery, T2WI sagittal plane and cross-sectional plane showed mixed high and equal signals and **g** T1WI showed a low signal in the deep incision. **h** serum C-reactive protein (CRP) level and erythrocyte sedimentation rate increase sharply from postoperative day 3. The SSI was confirmed by his complaint of localized pain, symptoms of fever, imaging test and laboratory

All patients who underwent PLF surgery with or without decompression for lumbar disk herniation, lumbar spinal canal stenosis and spondylolisthesis between January 2017 and July 2021 were reviewed and included in our study. The exclusion criteria were patients with (1) revision surgery; (2) combined cervical and lumbar fusion surgery; (3) non-contiguous segmental surgery; (4) drug treatment for cancer; (5) lack of postoperative information; and (6) congenital spinal deformity. The first consecutive patients (ERAS group) underwent elective lumbar fusion surgery from January 2019 to July 2021. These patients were then compared with the previous case-matched consecutive patients (non-ERAS group) who had undergone surgery from January 2017 to December 2018 before implementing the ERAS program. Demographic data including age, sex, body mass index (BMI), comorbidities and preoperative laboratory values were obtained before surgery. Intraoperative variables included the number of fused segments, estimated blood loss and surgical time. Postoperative variables included postoperative complications within 90 days after surgery, LOS, rate of 90-day readmission, rate of albumin transfusion and the total amount of albumin infusion. Postoperative serum albumin below 35 g/L was defined as hypoalbuminemia, and postoperative serum albumin below 30 g/L was defined as severe hypoalbuminemia. According to the Centers for Disease Control (CDC) and Prevention criteria, SSI was assessed was based on symptoms, histopathologic examination, imaging tests and bacterial cultures of the drainage [20]. Figure 1a–h provides an example of postoperative SSI following PLF surgery. Wound dehiscence (also known as poor wound healing) was diagnosed when the surgical wound’s superficial, partial or complete separation of surgical wound was recorded in wound nursing records. Persistent wound drainage refers to wound drainage for more than 3 days which created a moist dressing with a negative bacterial culture [4, 6].

### Statistical analysis

All statistical analyses were performed using the SPSS software (version 22.0; SPSS, USA). Continuous variables were expressed as mean and 95% confidence interval. Categorical variables were expressed as frequencies with percentages. Continuous variables were analyzed using a two-tailed Student’s *t* test, and categorical variables were analyzed using the Fisher’s exact or Chi-square tests. A *p* value of 0.05 was considered significant.

## Results

**Fig. 2:**
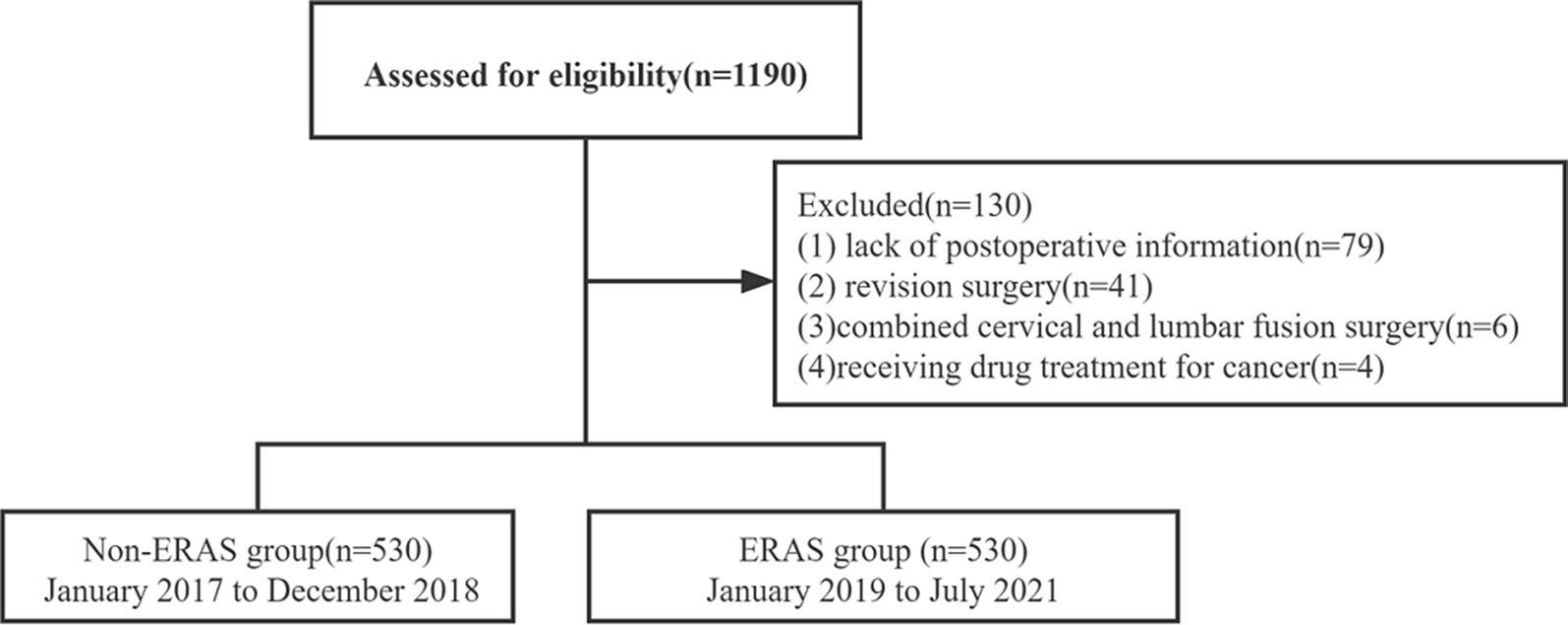
The study flowchart

We reviewed 1190 individuals who underwent PLF surgery between January 2017 and July 2021. Of these, a total of 1060 patients met inclusion criteria, and 130 patients were excluded because of lack of postoperative data, or history of combined cervical and lumbar fusion surgery. The study flowchart is shown in Fig. 2. A total of 530 ERAS patients were compared with the previous 530 patients who did not receive ERAS care. No significant differences in age, sex or BMI were identified between the groups, so further matching was not attempted. The mean age was 64.2 years in the non-ERAS cohort and was 65.0 years in the ERAS cohort (*p* = 0.27). More patients with old cerebral infarctions were in the ERAS group (*p* < 0.01), and other demographics and comorbidities were similar between both groups. No significant differences were observed in the number of fused segments and surgical time between groups. The serum preoperative albumin level of the ERAS group was similar to that of the non-ERAS group (39.6 g/L vs. 39.9 g/L, *p* = 0.31). Patients in the ERAS group had less intraoperative blood loss than those in the non-ERAS group; however, this was statistically insignificant (344.1 mL vs. 314.3 mL, *p* = 0.10) (Table 2).

**Table 2:** Baseline characteristics of patients in the two groups

Variable	Non**-**ERAS (n = 530)	ERAS (n = 530)	*P* value
Gender			*P* = 0.21
Female	323 (61%)	303 (57%)	
Male	207 (39%)	227 (43%)	
Age(yr)	64.2 ± 0.9	65.0 ± 1.0	*P* = 0.27
Weight(kg)	68.3 ± 0.9	69.5 ± 1.0	*P* = 0.09
BMI(kg/m^2^)	25.7 ± 0.3	26.0 ± 0.3	*P* = 0.21
Comorbidities			
Cardiovascular disease	288 (54%)	280 (53%)	*P* = 0.62
Diabetes disease	109 (21%)	134 (25)	*P* = 0.07
Mental disease	7 (1%)	10 (2%)	*P* = 0.46
Digestive disease	18 (4%)	21 (4%)	*P* = 0.63
Old cerebral infarction	7 (1%)	24 (5%)	*P* < 0.01^*^
Pulmonary disease	15 (2%)	10 (2%)	*P* = 0.31
Preoperative albumin	39.9 ± 0.3	39.6 ± 0.3	*P* = 0.31
Procedure-related			
Fusion level			*P* = 0.91
1–3	482 (90%)	483(90%)	
4–5	48 (10%)	47(10%)	
Operative time(min)	209.1 ± 30.2	203.0 ± 28.0	*P* = 0.16
EBL(ml)	344.1 ± 28.3	314.3 ± 21.1	*P* = 0.10
The rate of albumin infusion, n (%)	152 (29%)	39(7%)	*P* < 0.01^*^

BMI: body mass index; EBL: estimated blood loss

### Postoperative complications

Patients in the ERAS group had a lower incidence of postoperative wound-related complications than the non-ERAS group (12.4 vs. 17.8%, *p* = 0.02). The non-ERAS group had a significantly higher rate of wound dehiscence or poor wound healing (6% vs. 3%, *p* = 0.02); however, no differences were observed in postoperative SSI and persist wound drainage between both groups. Despite similar incidence of postoperative hypoalbuminemia (serum albumin less than 35 g/L) (50.4% vs. 45.7%, *p* = 0.12), the ERAS group had a lower incidence of severe postoperative hypoalbuminemia (serum albumin less than 30 g/L) (15.8% vs. 9.0%, *p* < 0.01). The albumin infusion rate (7.4% vs. 28.6%, *p* < 0.01) in the ERAS group was lower than the non-ERAS group (Table 3). Although fewer patients had postoperative cardiovascular complications, pneumonia and deep venous thrombosis, there were no significant differences in rates of these complications between groups (Table 3).

**Table 3:** The outcomes of patients in the two groups

	Non-ERAS (*n* = 540)	ERAS (*n* = 540)	*p* value
Wound complications, *n*(%) SSI, *n*(%) Wound dehiscence, *p* *n*(%) Persist wound drainage, *n*(%)	94 (17.8%) 21 (4.0%) 33 (6.0%) 40 (7.5%)	66 (12.4%) 16 (3.0%) 17 (3.0%) 33 (6.2%)	*p* = 0.02^*^ *p* = 0.40 *p* = 0.02^*^ *p* = 0.39
Other complications			
Cardiovascular disease	5 (1%)	5 (1%)	*p* = 1.00
Acute cerebral infarction	3 (0.6%)	3 (0.6%)	*p* = 1.00
Pneumonia	9 (1.7%)	6 (1.0%)	*p* = 0.44
Hematoma	6 (1.0%)	4 (0.8%)	*p* = 0.53
DVT	7 (1.2%)	5 (1.0%)	*p* = 0.56
Urinary tract infection	11 (2.0%)	4 (0.8%)	*p* = 0.07
Hypoalbuminemia Server hypoalbuminemia	267 (50.4%) 84 (15.8%)	242 (45.7%) 48 (9.0%)	*p* = 0.12 *p* < 0.01^*^
Albumin infusion	152 (28.6%)	39 (7.4%)	*p* < 0.01^*^
Preoperative LOS(d)	5.70.8	5.50.7	*p* = 0.32
Postoperative LOS(d)	9.51.7	8.01.5	*p* < 0.01^*^
Rate of readmission	34 (6.4%)	10 (1.9%)	*p* < 0.01^*^
Discharge to rehabilitation center, *n*(%)	22 (4.2%)	5 (1.0%)	*p* < 0.01^*^

SSI: surgical site infection; DVT: deep venous thrombosis; LOS: the length of stay

### The LOS and 90-day readmission

The preoperative LOS was similar between the groups; the average postoperative LOS in the ERAS group was 8.0 d, compared with 9.5d in non-ERAS group (*p* < 0.01). Compared with the control group, the ERAS group had a lower 90-day readmission rate (1.9% vs. 6.4%, *p* < 0.01). The ERAS group had a lower rate of discharge to rehabilitation center (4.2% vs. 1.0%, *p* < 0.01).

## Discussion

Wound complications are risk factors for prolonged LOS and higher hospitalization costs [2, 9, 21]. In addition to avoiding implant device-associated infections, perioperative management and nutritional support are essential to minimizing wound complications. In the present study, we found that our ERAS pathway was associated with a lower incidence of wound complications including SSI, wound dehiscence and persistent wound drainage. We also found that patients receiving the ERAS care had a lower rate of severe postoperative hypoalbuminemia without increasing the albumin transfusion. Patients in the ERAS group had shorter LOS and a lower readmission rate, consistent with previous studies [22, 23].

Despite advances in antibiotics and surgical instruments over the past few decades, wound healing following PLF surgery still remains a challenging clinical problem. Many studies identified risk factors and treatment for postoperative SSI [7, 18]; however, few reported other wound problem including wound drainage or wound dehiscence that might contribute to a reduction of satisfaction. In the present study, the rates of wound dehiscence and persist wound drainage were 4.5% and 6.7%, respectively, higher than the rate of postoperative SSI. Persistent wound drainage and wound dehiscence might be early symptoms of infection, and they could create a humid environment for bacteria growth. Equal attention should be paid to patients with noninfectious wound complications.

The ERAS protocol is a multidisciplinary and multifaceted perioperative care pathway, consisting of preoperative, intraoperative and postoperative interventions that may help to reduce the impact of those risk factors for adverse events [14, 24]. To the best of our knowledge, this is the first study to evaluate the impact of ERAS pathway on wound complications in Asian patients. Although no significant differences were observed for SSI and wound drainage, we found that the implement of ERAS was beneficial in reducing the incidence of wound complications (especially wound dehiscence) in patients undergoing PLF surgery.

Several possible reasons may explain our findings. First, previous studies showed that perioperative malnutrition and hypoalbuminemia were independent risk factors for postoperative SSI, and nutrition is critical for all wound healing steps (hemostasis, inflammation, proliferative and remodeling phase) [5, 25]. In the present study, the patients in the ERAS group had a lower incidence of severe postoperative hypoalbuminemia resulting from the nutrition support and early rehabilitation of digestive function [26]. Xu et al. [12] conducted a prospective randomized controlled trial and also found that perioperative multimodal nutritional management effectively reduced albumin infusion and incidence of wound drainage. Second, preoperative education on smoking and optimization of chronic disease may reduce wound complications. Pirkle et al. [21] retrospectively reviewed a PearlDiver national insurance claims database of 12,519 patients undergoing lumbar fusion surgery and found that diabetes was an independent risk factor for wound infection after single- and multi-level fusion surgery. In a systematic review and meta-analysis of 107 studies, preoperative smoking was found to be associated with an increased risk of the wound complications [27]. More efforts are needed to identify the effect of preoperative interventions on postoperative complications by conducting prospective randomized controlled studies. Finally, prolonged drain duration was proved to be associated with a higher incidence of wound infection in patients following lumbar spinal fusion surgery [7]. In the present study, patients in the EARS group were scheduled to have their drain removed on postoperative days 1and 2.

Nearly half of the patients had postoperative hypoalbuminemia or severe hypoalbuminemia, and a lower rate of severe hypoalbuminemia was observed in the ERAS group than the non-ERAS group. Avoiding mechanical bowel preparation and early postoperative enteral nutrition relieves irritation of the gastrointestinal tract and facilitates the recovery of gastrointestinal motility [28, 29]. These measures may have helped improve nutrition and reduce severe hypoalbuminemia (serum albumin < 30 g/L) in the ERAS group. However, the incidence of hypoalbuminemia (serum albumin < 35 g/L) was similar between groups; a more effective perioperative nutritional support protocol is needed to improve their nutritional status. A trend toward a lower rate of urinary tract infection in the ERAS group was observed (*p* = 0.07), although there was no statistical significance. A potential association might be detected in long-term studies with larger sample sizes. Moreover, the implementation of the ERAS pathway did not increase the risk of cardiovascular complications, acute cerebral infarction or local hematoma, consistent with previous studies on ERAS pathways [23, 30].

In the present study, although the preoperative LOS was similar between the groups, the postoperative LOS was 9.5 days in the non-ERAS group and 8.0 days in the ERAS group. We did not evaluate the effect of ERAS on postoperative LOS for short-segment and long-segment fusion surgery separately; however, previous studies had reported that patients in ERAS group had significantly shorter LOS than non-ERAS group after short lumbar fusion and long-segment deformity surgery [22, 24]. A lower rate of 90-day readmission was also observed in the ERAS group, consistent with a previous retrospective study of 124 patients conducted by Adeyemo et al. [14]. Postoperative multidisciplinary care and multimodal pain control may have contributed to the lower incidence of readmission for postoperative complications and transfer to rehabilitation centers.

However, this study had several limitations. First, it was a single-center study, and the patients included in our study were from our institution only. The variables data were acquired from our electronic medical records; therefore, we could not avoid the loss of partial information (e.g., total costs and patient satisfaction). The primary outcome of our study was the incidence of wound complications within 90 days; despite our efforts to identify wound problems, some minor wound complications might have been overlooked. Surgery-related variables, including blood loss and drainage volume, have also been reported to be associated with postoperative complications [31]; however, they were not included in the present study. We included consecutive patients who underwent elective PLF surgery performed by the same experienced team of surgeons, in order to avoid the effects of these confounding factors. The compliance with the ERAS protocol was associated with postoperative outcomes; however, due to the lack of a standardized perioperative management pathway, it was impossible to evaluate the compliance with the ERAS program for patients in the non-ERAS group; efforts are needed to maximize compliance with specific enhanced recovery pathway standards.

## Conclusion

In this retrospective study, we found that an ERAS pathway could help decrease the rates of postoperative wound complications and severe hypoalbuminemia following PLF surgery. Nevertheless, our ERAS pathway did not appear to reduce rates of other complications; therefore, more effective interventions are needed to improve postoperative nutrition. Additionally, it demonstrated that the ERAS pathway was associated with shorter LOS and lower 90-day readmission rates.

## Data Availability

The datasets analyzed during the current study are available from the corresponding author on reasonable request.

## Abbreviations

PLF: Posterior lumbar fusion
ERAS: Enhanced recovery after surgery
SSI: Surgical site infection
LOS: The length of stay
BMI: Body mass index
POD1: Postoperative day 1
POD2: Postoperative day 2
POD3: Postoperative day 3
DVT: Deep venous thrombosis
EBL: Estimated blood loss
